# A systematic mapping study on gamification within information security awareness programs

**DOI:** 10.1016/j.heliyon.2024.e38474

**Published:** 2024-09-26

**Authors:** Omid Pahlavanpour, Shang Gao

**Affiliations:** Department of Informatics, Örebro University, Örebro, Sweden

**Keywords:** Gamification, Information security awareness, ISA programs, Systematic mapping, Information security, Artificial intelligence

## Abstract

Information security awareness (ISA) has become a vital issue for organizations, as security breaches are usually attributed to human errors. ISP programs are effective ways to educate employees and enhance their information security knowledge. Gamification is a new concept in the area of ISA programs and it has been proven to be one of the most effective and proper ISA methods in both the private and public sectors. Despite a growing interest in employing gamification as an ISP program in recent years, there is a lack of study to provide a comprehensive overview of gamification within ISA programs and identify trends, patterns, and research gaps in this area in order to direct future research. To bridge this gap, a systematic mapping study is adopted as a research methodology. A total of 69 papers were selected and classified by document type, year of publication, research type, research contribution, gamification type, gamification in terms of adaptivity based on the target group, and gamification in terms of the use of artificial intelligence (AI) in order to make it user-tailored. The mapping study revealed that the published papers in this area were split between journals and conference papers with a higher proportion published in conference proceedings. Regarding the publication trend, from 2015 to 2022, gamification within ISA programs has come across to researchers' attention. The identified two main research types were evaluation research and validation research and the vast majority of the contribution type was tools. Moreover, content gamification has been used more commonly in ISA programs than structural gamification. Furthermore, the finding indicated that there were clear gaps in employing adaptive gamification, dynamic adaptive gamification and AI-based adaptive gamification, which makes these areas significant for future research.

## 1 Introduction

Information security, AKA ‘InfoSec’, refers to the protection of various types of information from unauthorized access and use, with the aim is to provide confidentiality, integrity, and availability [1]. Cyber security often comes in the same category as information security with the distinction that cyber security is not just limited to the protection of information assets, it also considers other assets such as the individual him/herself [2].

If sensitive information of organizations is disclosed, damaged, or lost during security incidents, not only would organizations face severe economic damages imposed by law and regulations (e.g., GDPR), but also it could have a negative impact on organizations' reputation and customers' confidence in organizations. According to Cybersecurity Ventures, the cost of cybercrime is expected to reach $10.5 trillion annually by 2025. This shows the great importance of information security in today's digital society.

Social engineering, ransomware, and lack of awareness are the topmost InfoSec related threats reported in Chapman [3]. This implies that human error and lack of knowledge are fundamental problems. ENISA [4] also indicated that about 77 % of the companies' data breaches were due to exploitation of human weaknesses. Human errors whether directly and/or indirectly are the main sources of the majority of security incidents including both intentional and unintentional misbehavior [5]. To prevent security breaches, it is not possible to just focus on technical controls. It is also vital to consider formal and informal controls that focus on processes (e.g., guidelines, procedures) and people (i.e., employee's security awareness) [6]. A large number of information security breaches are caused by employees [7]. For instance, employees may unintentionally grant unauthorized individuals access to sensitive systems by failing to log out of their accounts. The human factor is one of the most common reasons for security breaches in organizations due to the employees' lack of information security awareness [8,9]. It is essential for users to be educated in the area of InfoSec. They should adopt a behavior that is responsible for protecting sensitive information and supporting security. To achieve this, information security awareness programs should be employed in organizations [10].

Information security awareness (ISA) is defined as the individual's passive involvement to increase their interest in InfoSec issues [11]. Information security awareness plays a critical role in organizations' settings, such as safeguarding sensitive data, ensuring security policy compliance, and mitigating security threats. Furthermore, an ISA program is a process that aims to change individuals' perceptions, values, attitudes, behavior, norms, work habits, and organizational culture and structures concerning secure information practices [12]. This definition emphasizes the social aspect of ISA as an organized and ongoing attempt to guide the behavior and culture of an organization with respect to information security issues [13,14].

Given the evolution of technology, learning environments should also be designed to accommodate the needs of modern learners and provide a motivating and conducive learning setting. To address this, the use of gamification including games and game-like environments could be a proper solution [15]. *“Gamification is the use of game design elements in non-game contexts”* [16]. Gamification contains a fun element, and is able to offer an engaging and interactive way to deliver educational programs to learners (e.g., Moll and Gao [17]). Since ISA programs can be seen as educational programs, gamification could make a substantial contribution to users' active participation in ISA programs, an improved interactive learning approach in ISA programs, and an enhanced culture of ISA within organizations. For instance, social media plays an important role in people's daily lives today. Many people use social media to share a variety of content (e.g., documents, photos). While social media provides valuable digital communications and networking opportunities to users, it also can be used to create cyber-attacks [18]. Thus, individuals should be aware of security threats that could be introduced by social media. Gamification (e.g., gamified online quizzes) can also make the learning process more interactive and engaging [19]. For instance, gamified online quizzes can be applied to ISA programs to help learners understand information security best practice in an interactive manner. Therefore, it is important to conduct a review study to gain a better understanding of gamification within ISA programs.

There has been an increasing interest in gamification within ISA programs in the past couple of years. Gamification has been proven to be one of the most effective and proper ISA methods in both the private sector and the public sector [13]. It allows employees to play and learn about various security threats and vulnerabilities. For instance, Ghazvini & Shukur [20] designed a serious game (i.e., InfoSecure) to improve Information Security Awareness (ISA) in the healthcare sector. According to their evaluation results, employees' ISA levels have relatively increased after playing the game. Moreover, gamification has been employed in ISA programs to educate users within the InfoSec context and hence improve their learning outcomes. The role of gamification in this context is to increase both experiential outcomes and instrumental outcomes [21]. Tan, Sunar, and Goh [22] demonstrated that the introduction of gamification in education could lead to good educational results. And Matovu, Nwokeji, Holmes, and Rahman [23] found that gamification is an effective technique for knowledge acquisition in cybersecurity awareness in smaller universities and colleges. In addition, some studies explored the use of gamification to enhance users' engagement and motivation and ultimately improve their knowledge with regard to InfoSec issues. For instance, Chen, Zhang, Zhang, and Lyu [24] examined the effect of gamified InfoSec education systems on improving users’ ISA and protection behavioral intention.

Previous literature studies on ISA tend to focus on ISA methods and factors (e.g., Khando et al. [13]). According to the findings above, gamification plays an important role in improving users' ISA, and gamification has been identified as one of the most effective ways to improve the efficiency of ISA programs which plays an important role in improving users’ ISA. However, there is a lack of studies on a holistic structured view of the state of the art in gamification within ISA programs. Therefore, further research is needed to advance the understanding of gamification within ISA programs.

This study aims to provide an overview of the research concerning gamification and ISA programs and to identify trends, patterns, and research gaps in this area in order to direct future research. To address this, we conducted a systematic mapping study [25,26], which involves searching the literature to verify the nature, trend and extent of studies published in the area of interest. A comprehensive overview of gamification within ISA programs would provide a road map directing future research.

The study would provide the following contributions. Firstly, it would help researchers who need a reference on where and how to initiate their studies on gamification within ISA programs. Secondly, it would provide some insight for information security practitioners and managers who faced problems in their ISA programs and are looking for possible solutions. Thirdly, it would offer inputs for system developers working on designing gamification within ISA programs. Lastly, it would be of help for users to gain a better understanding on the current specification of gamification within ISA programs and its pros and cons.

The rest of this paper is organized as follows: Section 2 presents the background of the research including the definition of major concepts and theories used in this study. The third section defines the research questions, and details the chosen research methodology. Section 4 presents the results. Section 5 discusses the outcomes of the research and presents the threats to the validity of this study. Lastly, section 6 concludes this paper.

## 2 Research background

### 2.1 Information security awareness programs

The purpose of an ISA program is to raise awareness regarding information security, explain rules and define proper behaviors needed to use information technology systems, and provide employees with the necessary skills and expertise to work securely [27]. According to NIST [27], there are a variety of topics when it comes to developing ISA material. Some of these topics are password usage and management, social engineering, unknown e-mail/attachments, handheld device security issues, incident response, etc.

There are various methods to conduct ISA programs. Khan et al. [28] categorized these methods into seven types namely educational presentation, e-mail messaging, group discussions, newsletter articles, video games, computer-based training (CBT), and posters. Their study shows not only should ISA methods increase the InfoSec knowledge of the participants but also they should have the ability to change users' InfoSec behavior [28]. Albrechtsen and Hovden [29] discussed and evaluated the effects of an ISA program by involving users directly. The results indicated that the intervention method produced stronger changes in employees’ attitudes and knowledge of InfoSec than the mainstream ISA measures (e.g., emails) [29]. Furthermore, Cohen et al. [30] developed ConGISATA - a continuous gamified ISA training and assessment framework based on embedded mobile sensors. And Gasiba, Lechner, Pinto-Albuquerque, & Porwal [31] introduced a new automatic challenge evaluation method together with a virtual coach to improve existing ISA training programs.

Gamification has emerged as a growing area of interest within ISA programs. It aims to apply game features such as points in non-game context to encourage participants to perform a task or set of tasks in order to promote their InfoSec knowledge and behavior [32]. Although gamification has great potential to improve the performance of ISA programs in areas where current efforts are not succeeding, it also has significant pitfalls that should be considered in its design and application [33].

### 2.2 Different types of gamification

Gamification has been widely used in education and training to improve the learning processes and outcomes [34]. Gamification in learning and education can be defined as *“a set of activities and processes to solve problems related to learning and education by using or applying the game mechanics”* ([35], p. 29). The goal of gamification is to encourage the engagement and motivation of users (e.g., Ref. [36]) and make a beneficial change in users' behaviors [37].

Gamification in general has been divided into two categories namely structural gamification and content gamification [38]. In structural gamification (i.e., gamifying the content), the content itself does not become the game instead it presents in a game-like way. Structural gamification is mostly composed of a template-based approach and it is time and cost-efficient [38]. Structural gamification could be either in digital forms such as computer games or physical forms like tabletop games. Content gamification, which often is known as a serious game, is another type of gamification where the content is turned into a game. It requires more time and investment to develop the game. Once developed, it can be only used for that specific learning objective [38]. A serious game is a kind of game developed for a purpose (e.g., training [39]) other than entertainment [40].

### 2.3 Personalizing gamification within ISA programs

Flores & Ekstedt [41] found that personalizing InfoSec training content and combining them with practical exercises could make it more understandable and relevant to the audience to improve their ISA. Therefore, to enhance the effectiveness of ISA programs, these programs need to be tailored to address specific groupings of employees within the organization [10]. The same applies to gamification within an ISA program. Gamification should be tailored to the target users.

Adaptive gamification is a novel and rapid-growing research trend. It aims to improve traditional gamification approaches and transform them into user-centered and personalized ones coupled with specific characteristics of different users and contexts [42]. Thus, it is important to have a good understanding of users’ characteristics and needs to help designers individualize gamification concepts within ISA programs [43]. To achieve this, theories (e.g., the self-determination theory [44]) and frameworks (e.g., the gamification user types hexad [45]) can be used. For instance, self-determination theory states that people tend to grow by their intrinsic psychological needs, which can be influenced by various social and cultural factors. According to self-determination theory, three components are associated with intrinsic motivation: autonomy, competence, and relatedness (Deci & Ryan, 2002). Moreover, the Gamification User Types Hexad [45] is a decent framework for user classification that can be employed for gamification research.

People's preferences and features are dynamic in nature and could change over time [46]. Studies also observed the problem of the decline in engagement and the loss of interest among users in gamification over time [47]. To address this problem, it is important to continuously re-engage participants [47]. Therefore, adaptive gamification should not be a one-time process that involves changing gaming features to accommodate the needs of users. Instead, it needs to be a continuous process that continuously adjusts the gamification elements to meet the needs and preferences of the users. Previous studies (e.g., Alomair & Hammami [15]; Böckle et al. [42]; Passalacqua et al. [48]; Thiebes et al. [47]) indicated that having a dynamic approach to adapting gamification could provide a more user-centered training that has better performance and outcomes. Hence, it is important to continuously receive feedback from the employees and assess their performance to efficiently adapt the gamification to their needs and capabilities within ISA programs [33].

### 2.4 The use of artificial intelligence (AI) in gamification within ISA programs

Haenlein & Kaplan [49] define AI as *“a system's ability to correctly interpret external data, to learn from such data, and to use those learnings to achieve specific goals and tasks through flexible adaptation”*. Machine learning (ML) is the subset of artificial intelligence defined as the process for the development of computer algorithms to transform data into intelligence [50]. Machine learning is a set of techniques and practices that assist humans in various decision-making and analysis tasks by providing valuable information [51].

Machine learning has a significant role in enhancing the degree of adaptivity in gamification [42]. The use of machine learning methods has gained increasing popularity in gamification because it has a great ability in tailoring the gamified interactions and configuring the interaction parameters dynamically [51]. Machine learning models have been used to optimize gamified learning platforms. For instance, Lopez & Tucker [52] developed a machine learning model to predict an individual's performance on a gamified task with an accuracy of 76 percent. This model could help game designers develop more effective and engaging gamified applications by allowing them to consider task characteristics and individuals' facial expressions [52].

The integration of AI into gamification in ISA programs could be a proper solution to make gamification adaptive and maintain its adaptiveness based on the target group [43,51]. AI-based adaptive gamification would be helpful to improve users’ engagement and motivation to enhance the efficiency of ISA programs.

### 2.5 Related works

Khando et al. [13] conducted a systematic literature review to explore ISA methods and factors used for enhancing employees’ ISA within both the private and public sectors. They demonstrated that one of the most effective ways to improve the efficiency of ISA programs was through gamification. Moreover, Sharif & Ameen [53] conducted a literature review with the aim of investigating which InfoSec training methods are adopted for raising cyber security awareness, and to what extent these methods are effective. The results show although gamification has a positive impact on users' motivation to improve their ISA, there is a lack of a well-defined structure in gamification within the ISA programs context. Furthermore, Lindberg [54] investigated the relationship between game elements, users, and risky behavior in the context of ISA programs to identify which game elements have a positive/negative impact on the users. However, they could not draw a conclusion due to the lack of a gamified system within the ISA programs context.

Although a growing interest has been noticed in gamification within ISA programs in recent years, the previous reviews have been limited to the benefits and challenges of gamification and its impact on ISA of employees and there is no holistic structured overview of gamification within ISA programs. Therefore, it would be interesting and help with future research directions to provide an overview of the current status of research in this area, which is the aim of this systematic mapping study.

## 3 Research methodology

**Fig. 1:**
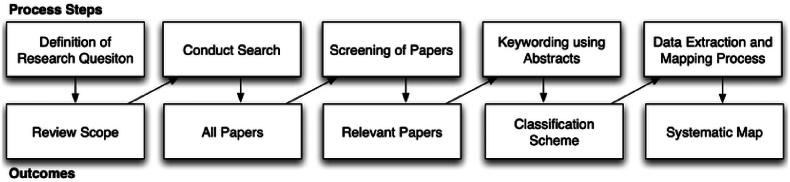
The Systematic Mapping Process [26].

This study aimed to provide an overview of the research concerning gamification and ISA programs and to identify trends, patterns, and research gaps in this area in order to direct future research. To achieve this, a systematic mapping study [25,26] was carried out to explore studies related to gamification within ISA programs. A systematic mapping study is a research method that can be used to provide a broad overview and classification of existing literature on a particular topic or research area. It aims to systematically collect, categorize, and analyze existing literature to identify trends, gaps, and patterns within the research field. In this study, we follow the process of systematic mapping study proposed by Ref. [26]. Fig. 1 depicts the mapping process workflow and all the phases. It includes five steps, each with a corresponding outcome.

### 3.1 Research questions

The principal goal of this study is to analyze publications concerning gamification within ISA programs to present a comprehensive overview of this topic area. Six research questions have been chosen to structure the overview. Table 1 illustrates the research questions along with their associated motivation.

**Table 1:** Research questions and Motivation.

Research Questions	Motivation
RQ1. Which document types are the main targets for research in the area of gamification within ISA programs?	To identify where the research in the area of gamification within ISA programs is more likely to be found as well as potential targets for the publication of future studies.
RQ2. How have publication numbers in the domain of gamification within ISA programs evolved over time?	To identify the trend of publication frequency concerning the subject under study over time.
RQ3. What are research types and contribution types proposed in research concerning gamification within ISA programs?	To explore the different types of research and various kinds of contributions reported in the literature in the field of gamification within ISA programs.
RQ4. What are the types of gamification used in ISA programs?	To provide an overview of gamification types used in ISA programs which is one of the foundations of the systematic mapping study
RQ5. What is the status of gamification in terms of adaptivity based on the target group in research concerning gamification and ISA programs?	This question aims to provide an overview of gamification in terms of adaptivity based on the target group in ISA programs in order to identify possible gaps and pave the path for further research.
RQ6. What is the status of the use of AI in research concerning gamification and ISA programs in order to make gamification user-tailored?	AI or more specifically machine learning has proven to be a proper solution in personalizing gamification. The objective of this question is to provide an overall view of the use of AI in research in the area of gamification and ISA programs and display the current trends.

### 3.2 Conducting the search

The search was conducted in March 2022. The following two databases have been used for the literature search: Scopus and the Web of Science. We used four keywords that are relevant to our research topic (i.e., gamification, game, gamified, and security awareness) and performed different combinations among them to conduct the search. The rationale behind the use of the aforementioned keywords is as follows. On the one hand, since both serious games, and the use of gamified elements in non-game contexts are considered gamification [38], we employed these three keywords (gamification, gamified, and game) to cover a broad range of gamification papers. On the other hand, there are several synonyms for ISA programs (e.g., ISA campaigns, ISA methods, information security training) [2]. We omitted ‘program’ and ‘information’ from the ISA programs phrase and opted for security awareness as this keyword could retrieve more papers in the context of ISA programs. In this way, we could avoid the restriction in search strings and extract a broad range of research in the area of gamification within ISA programs. This was of help to have a complete map and mitigate the risk of bias [26]. Furthermore, we also carried out a backward citation search in order to retrieve more papers that are relevant to our study. The result of the backward citation search added 16 more papers to our study. The final result of this stage was a set of 1499 papers. Table 2 illustrates the conducted searches.

**Table 2:** The conducted searches.

No.	Database	Keyword 1	Operator	Keyword 2	Result
1	Web of science	“gamification”	And	“security awareness”	21
2	Web of science	“gamified”	And	“security awareness”	5
3	Web of science	“game”	And	“security awareness”	52
4	Scopus	“gamification”	And	“security awareness”	256
5	Scopus	“gamified”	And	“security awareness”	103
6	Scopus	“game”	And	“security awareness”	1046
7	Backward citation search				16
Sum					1499

### 3.3 Screening of relevant papers

The aim of this selection is to identify those papers that are most relevant to the objective of this mapping study. The first screening step is to remove duplication of the retrieved papers from the initial search. The next step is to identify the relevant papers by employing our inclusion and exclusion criteria shown in Table 3. After that, every paper retrieved from the previous step was reviewed carefully based on its title, abstract, keywords, and conclusions to identify the more relevant ones. Lastly, the remaining papers were read in their entirety to include the most relevant papers. As a result, 69 papers were selected for this systematic mapping study. Table 4 shows the number of selected studies per phase.

**Table 3:** Inclusion and exclusion criteria.

Inclusion criteria	Exclusion criteria
The paper is in the area of gamification and ISA programs.	Studies not written in English
The full text of the paper is available	The full text of the paper is not available
Only documents that their type is journal article or conference paper	Other document types such as reviews, book chapters, books, conference reviews, editorials, and notes are not included
Only primary studies	Papers that clearly expressed that the main output of their study is a systematic literature review or mapping study

**Table 4:** Overview of selected studies per each step in the screening process.

Total numbers of papers from the initial search	After removing duplication and applying inclusion and exclusion criteria	After reading the title, abstract, keywords, and conclusions	After reading entirety
1499	278	91	69

### 3.4 Classification scheme

The goal of the data extraction strategy is to provide a comprehensive answer to each of the RQs. The relevant classification scheme(s) for answering each RQ is presented below.

RQ1: To find the answer to this question, document types for each identified paper have been determined.
RQ2: To explore the publication trend, papers have been categorized based on the year of publication.
RQ3: For exploring and categorizing research types, we employed the scheme proposed by Wieringa et al. [55]. It consists of six categories as follows:
• Proposal of solution: The paper proposes a solution for a particular problem and discusses its relevance, without a complete validation. The solution technique should be novel or at least significantly improved over an existing technique [55].
• Validation research: The objective of the paper is to investigate the properties of a proposed solution that has not been implemented in practice. The possible research methods for conducting this type of study are experiments, simulation, prototyping, etc. [55].
• Evaluation research: The aim of the paper is to investigate a problem or implement a technique in practice. The novelty of knowledge made by the paper is an important criterion. It also shows the result of the implemented technique in terms of benefits and drawbacks. Possible research methods are case study, field study, field experiment, survey, etc. [55].
• Philosophical papers: “*These papers sketch a new way of looking at existing things by structuring the field in the form of a taxonomy or conceptual framework”* [55].
• Opinion papers: The paper provides the personal opinion of the author about a particular technique or idea, without relying on related work and research methodologies. For instance, whether the technique is good or bad, or how things should be done [26].
• Experience papers: An experience paper is a type of paper that describes how something was performed in practice. It should be the author's own experience [26].

Classification criteria (research type): To increase the quality of our classification and to choose the best possible category for each paper we followed questions proposed by Wieringa et al. [55] and keywords suggested by Zapata et al. [56]. Papers can be categorized into multiple categories. For instance, it is possible to write a paper that proposes a new technique and then present a sound validation of the technique, at the end the author discusses his or her opinion regarding what other researchers should do [55]. However, for this study, we decided to see what the major part of the paper talks about and then put the paper just in one of the associated categories.

Categories for contribution type were extracted from Ouhbi et al. [57] and Engström & Runeson [58]. The categories were slightly adapted and merged together to fit our study, for example, guidelines and open items were combined together and metrics were removed from our list.

• Tool: Anything used as a means to accomplish a task or purpose.
• Method: A means or manner of procedure that consists of several steps that should be carried out for acquiring the research scope or knowledge.
• Model: A representation of a system that enables the investigation of particular attributes.
• Framework: A conceptual or real structure that acts as a guide or support for the creation of something useful.
• State of Knowledge: *“Summary of the existing body-of-knowledge of the domain”* [59].
• Guideline/Open item: An indication of the procedure with which a course of action can be specified or a sign of identified issues that need to be addressed.

Classification criteria (contribution type): it is possible that one paper has more than one research contribution. In this kind of situation, we count each contribution separately.

RQ4: in order to answer this question, the gamification classification proposed by Dubey [38] has been adopted.
• Structural gamification: In structural gamification, the content itself does not become the game instead it presents in a game-like way.
• Content gamification: In this type of gamification, the content is turned into a game.
• None: No gamification as a tool is proposed in the paper. Either structural gamification or content gamification could be considered in the paper.
RQ5: To find the answer to this question, we extracted the categories from Alomair & Hammami [15] and Göbel & Wendel [60]. They were slightly adapted to fit our study as follows:
• Non-adaptive gamification: Non-adaptive gamification, also referred to as standard gamification, is a “one size fits all” approach that overlooks the diversity of context and users' needs, abilities, and preferences [15,61].
• Adaptive gamification: Adaptive gamification is user-centered gamification that takes into account different users' personalities, needs, and values [42]. Everything relevant to a game can be adapted to make it more engaging. This includes visual and optical appearances, audio, and other elements such as the game storytelling, the game difficulty, the content generation, the guidance or hinting on the goals, etc. [60].
• None: The paper does not provide any gamification as a tool to educate users or any framework or methods as means for designing adaptive gamification.
RQ6: In order to answer this question, we defined the categories based on Scholefield & Shepherd [62] and adapt them slightly to fit our study:
• AI-based adaptive gamification: The paper employed artificial intelligence, more specifically machine learning, to make the gamification adaptive based on the target group [43].
• Non-AI-based adaptive gamification: The paper did not use AI to build or design personalized gamification [43].

### 3.5 Data extraction

During the data extraction phase, we extracted the information needed from each of the included articles to help us address our research questions and sorted the information into a data extraction form. The relation between data item and RQ can be found in Table 5. The filled data extraction form can be found in Appendix A.

**Table 5:** The relation between data item and RQ.

Data Item	RQ
Title	N/A
Document Type	RQ1
Reference Information (Including Year Information)	RQ2
Research Type	RQ3
Contribution Type	RQ3
Types of gamification	RQ4
Adaptive gamification/Non-adaptive gamification/None	RQ5
AI-based gamification or Not	RQ6

## 4 Results

This part presents the results of the research questions described in Table 1.

### 4.1 Which document types are the main targets for research in the area of gamification within ISA programs(RQ1)?

**Fig. 2:**
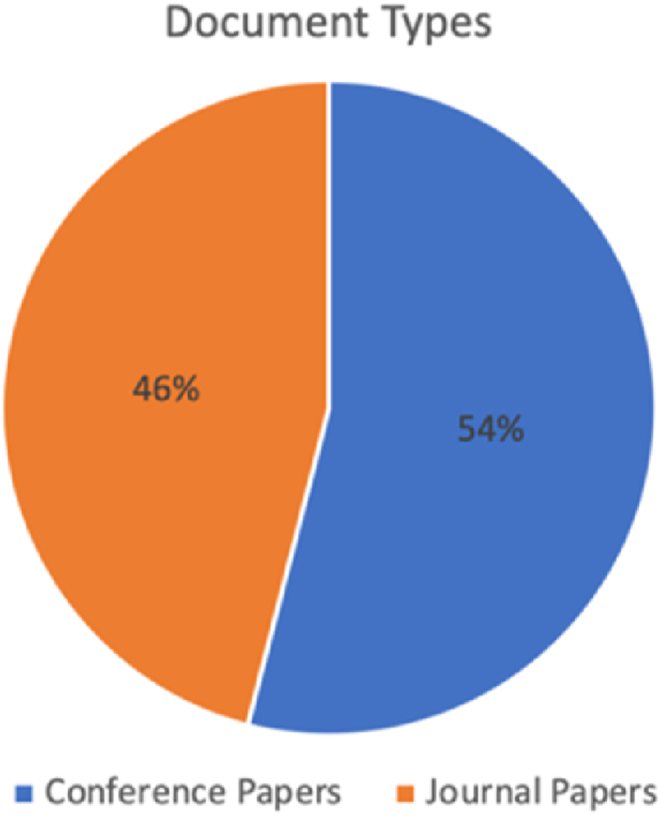
The ratio of document types among selected papers.

Fig. 2 illustrates the number of papers published based on the document type. 37 papers out of 69 papers were in the conference paper format (54 %) while the rest (i.e., 32 papers) were published in the journal paper format (46 %). It is noteworthy that the journal (i.e., Computers and Security) has published four papers in this research field, the highest number among all the targeted publication venues.

### 4.2 How have publication numbers in the domain of gamification within ISA programs evolved over time (RQ2)?

**Fig. 3:**
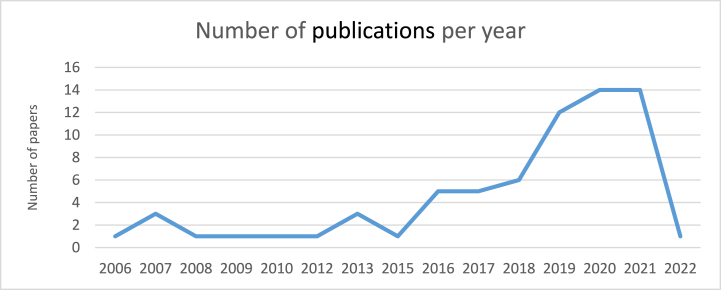
Number of papers published per year.

Fig. 3 illustrates the number of papers published per year. From the year 2006–2015, the number of publications experienced an almost steady trend with little fluctuation. However, from 2015 onwards the publication rate increased rapidly until 2022. The dramatic decrease in 2022 can be explained due to the time of conducting this study and it does not reflect the real number of papers in 2022 since the data extraction phase was performed in May 2022. The years (i.e., 2020 and 2021) have a maximum rate of publication (20.29 %) and the years 2006, 2008, 2009, 2010, 2012, and 2015 have a minimum rate of publication (1.45 %).

### 4.3 What are research types and contribution types proposed in research concerning gamification within ISA programs?

**Fig. 4:**
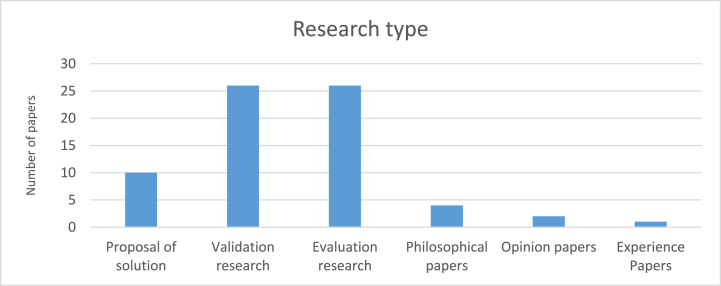
Number of papers published per research type.

We classified research types according to the research type facet defined in section 3.4. The mapping result is as follows: proposal of solution (10), validation research (26), evaluation research (26), philosophical papers (4), opinion papers (2), and experience papers (1). According to Fig. 4, validation research and evaluation research were the most commonly employed research types by authors, while opinion papers and experience papers were the least utilized.

In evaluation research and validation research, the authors evaluated the proposed solution to the problem to check its applicability, usability, benefits, and drawbacks. The difference is that validation research is performed mostly through prototyping without implementation, but in evaluation research, the authors implemented the solution in a real-world project. For instance, Newbould & Furnell [63] conducted validation research and developed an awareness-raising game to educate users about social engineering attacks in an interactive way. Then they tested the initial prototype of the game with 21 participants to observe its outcomes [63]. Ghazvini & Shukur [20] conducted evaluation research and developed a serious game as an information security awareness program for the healthcare industry. To evaluate the game's performance and observe its effects on employees, they implemented the game at a selected healthcare organization [20].

Proposal of solution papers mainly attempted to recover human parts from security threats, especially phishing attacks. Their solutions mostly were in the form of designing games such as Chen et al. [64] or creating a framework to develop a game such as Blythe & Coventry [65]. Philosophical papers mainly provide a taxonomy of various types of phishing attacks and their associated InfoSec training methods such as gamification as a solution [66,67]. We found two opinion papers. The first one proposes how to combine education and technology like interactive video games to reduce social engineering risks and damages [68], and the second one suggests how organizations deal with the personal information of employees when conducting ISA educational games to prevent severe consequences of the General Data Protection Regulation (GDPR) [69]. The only experience paper in our list (i.e., [96], [70]) presented some ideas based on the authors' industry experience and observations to address two issues, a). how to select proper secure coding guidelines, and b). how to enhance the ISA of software developers about secure coding based on these guidelines.

**Fig. 5:**
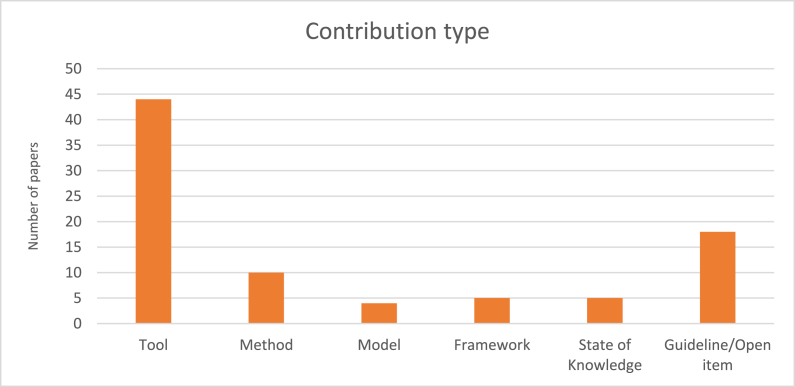
Number of papers published per contribution type.

According to Fig. 5, it was revealed that most of the solutions to the lack of users' InfoSec awareness were tools, i.e., the use of gamification as an ISA tool to educate users. For instance, Denning et al. [71] designed and produced a recreational tabletop card game called Control-Alt-Hack for increasing computer security awareness. The major InfoSec topic addressed in these educational tools was phishing attacks. There are also a few papers about other topics such as password policy [72], physical security [73] and secure coding [31]. Moreover, ten papers include methods. For instance, Kritzinger [74] offered a game-based method that can be carried out to enhance the cyber-safety awareness of participants. In addition, five papers proposed a state of knowledge. For instance, Alhashmi et al. [66] indicated that *"a well-designed game-based training delivery method can potentially offer quick learning and proficiency in cybersecurity fundamentals"*.

Furthermore, five papers presented frameworks that depicted how to design and build serious games and gamification elements to raise ISA. For instance, Blythe & Coventry [65] created a new framework, based upon literature findings, for game design that enhances the InfoSec behavior of end-users. And models were the least contribution type. For instance, Luh et al. [75] offered PenQuest, a gamified model designed to provide a holistic view of information security attacks and their mitigation. Their model defines and takes into account a wide range of actors, assets, and actions in order to enable the cyber risk assessment.

**Fig. 6:**
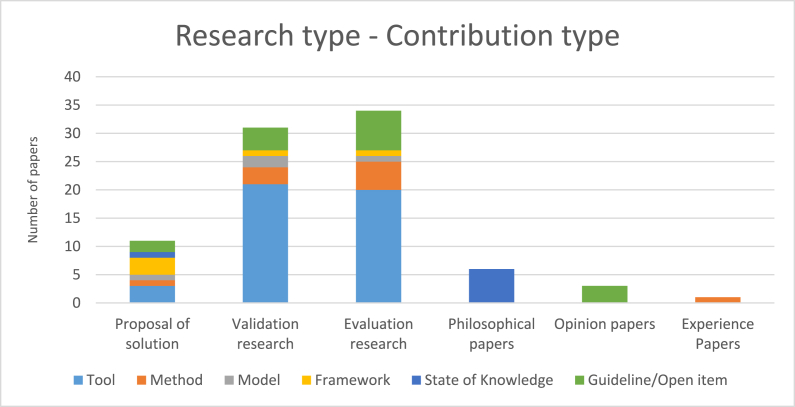
Number of papers published per research type categorized by contribution type.

Fig. 6 illustrates the number of papers published per research type categorized by contribution type. This enables us to monitor what contribution types are involved in each research type facet. The most contribution to the validation research and evaluation research were tools. In the proposal of solution research, we observed a diversity of contributions. Philosophical papers offered states of knowledge while opinion papers presented guidelines/open items.

### 4.4 What are the types of gamification used in ISA programs?

**Fig. 7:**
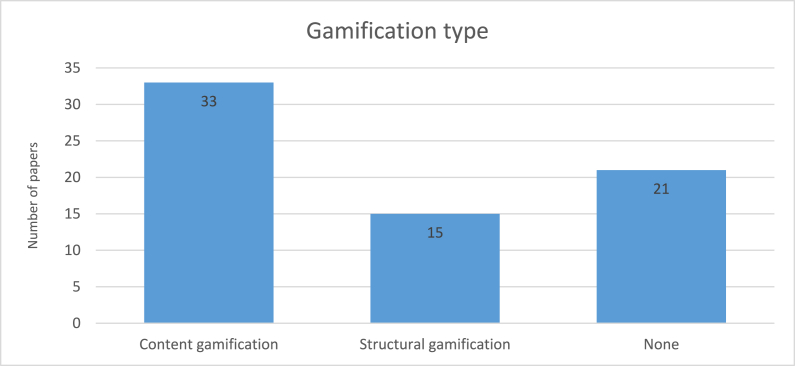
Number of papers published per gamification type.

According to Fig. 7, content gamification (47.83 %) had more share than structural gamification (21.74 %) in our retrieved papers. And it is interesting to note that among all the included papers, 21 papers did not propose gamification, either as content gamification or structural gamification. As one example of content gamification type paper, Arachchilage et al. [76] designed and developed a mobile game as an InfoSec educational tool that helps computer users protect themselves against phishing attacks.

Furthermore, Scholefield & Shepherd [62] employed gamification techniques to develop a role-playing quiz application (RPG) on the Android platform for educating average users about password security. This game falls into the structural gamification category because it can be used for other InfoSec topics just by changing the questions. Indeed this application is a simplistic multiple-choice quiz, but with the added features of gamification to motivate users and improve their performance without turning the content (password security) into the game. These gamification features include a specific theme (RPG-style game with characters), on-screen progress/feedback (the health bar per character), time pressure (timer), consequences (if the user is incorrect, they lose health points), and competition (by means of a leaderboard). The authors also mentioned that another version of the game to cover various kinds of topics such as phishing and information sharing would be developed in the future.

### 4.5 What is the status of gamification in terms of adaptivity based on the target group in research concerning gamification and ISA programs?

**Fig. 8:**
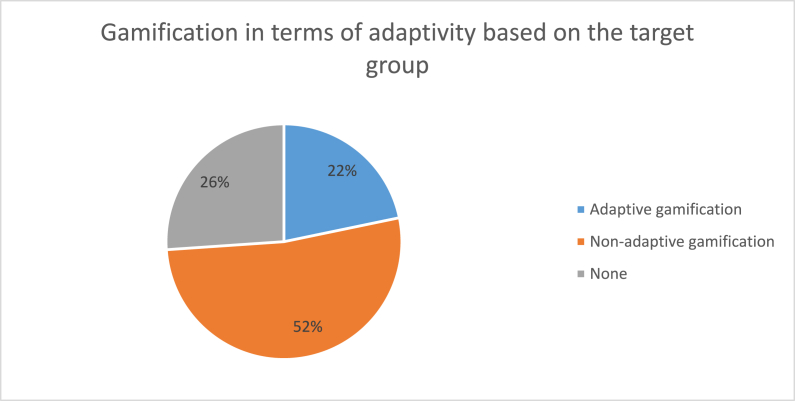
The ratio of gamification in terms of adaptivity according to the target group.

According to Fig. 8, only 15 papers (21.74 %) were classified as the adaptive gamification category. These papers include studies that made their gamification tool adaptive based on the target groups in addition to studies that proposed frameworks or methods for designing adaptive gamification. To better understand the papers included in the adaptive gamification category, some representative examples are provided. Goeke et al [77] employed one of the existing online serious games and enhanced it to the new version to train employees and cover their vulnerability to social engineering attacks. The new game (i.e., Protect) focuses on having highly configurable game settings and content to enable adaptive gamification according to the player's skills and context as well as the ability of the integration into training platforms. For instance, the difficulty of the game is controllable if the player's knowledge improves or the game scenarios can be adapted if the employee changed his/her department and faced new threats in the new department [77].

Furthermore, Ki-Aries & Faily [78] devised an iterative method for identifying security-related human factors by integrating personas into the design and implementation of ISA programs such as gamification. This method consists of 6 ongoing steps namely needs and goals, personas, analysis, design, development, implementation, and review. The goal of the second step (i.e., personas) was to extract relevant information from employees explaining behaviors and perceptions relating to the business and information security. This step was performed by interviewing employees randomly to gain insights into their roles and experiences in their workplace and to identify relevant characteristics and needs [78]. This method can be carried out for designing and developing user-tailored gamification (adaptive gamification).

**Fig. 9:**
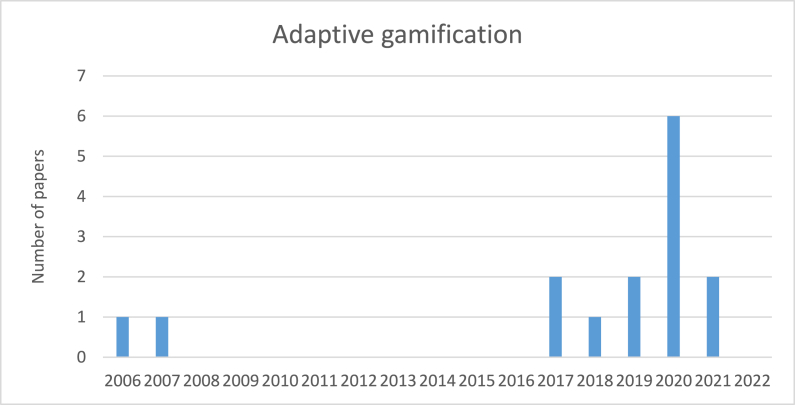
The number of papers concerning adaptive gamification per year.

Fig. 9 illustrates the number of papers that considered adaptive gamification per year. From 2006 to 2017, only 2 papers were labelled in the adaptive gamification category. But an increased rate was observed in this category from 2017 onwards. The maximum number of publications was the year of 2020 with 6 papers.

Among papers included in the adaptive gamification facet, we came across a few numbers of papers that employed continuous adaptive gamification (e.g.,Ghazvini & Shukur [20]; Gjertsen et al. [33]; Hatzivasilis et al. [79]; Mittal et al. [80]). For instance, Hatzivasilis et al. [79] aimed to support the development of dynamic training procedures by combining cyber-security modeling and pedagogical practices. First, the gamification program was tailored to the trainee's needs, and afterward, the adaptation process would continue based on his/her performance.

In the list of papers classified as non-adaptive gamification, there are several papers that after conducting gamification as an ISA program, conclude that some parts of their gamification program need to be refined and consequently adapted based on the target audience and context [62,71,81,82]. For instance, González-Tablas et al. [82] introduced Crypto Go, a physical card game to enhance users' knowledge of cryptography. After the initial evaluation of the game, the author realized that some improvements are necessary for the subsequent version of the game including adapting the game content based on the mathematical knowledge of the target audience to maximize the game efficiency and usability.

### 4.6 What is the status of the use of artificial intelligence in research concerning gamification and ISA programs in order to make gamification user-tailored?

**Fig. 10:**
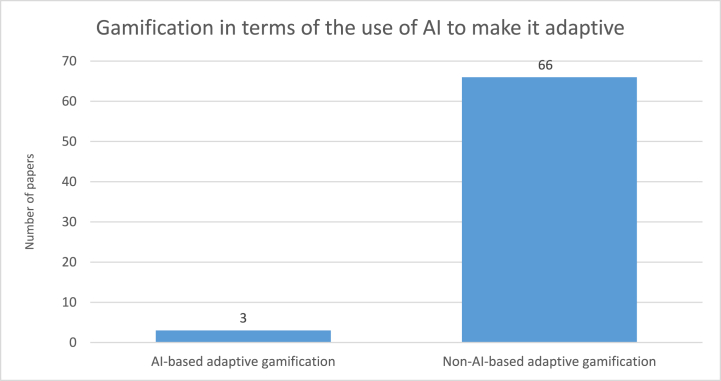
Number of papers using AI to make gamification user-tailored.

According to Fig. 10, Fig. 3 out of 69 papers (4.35 %) fall into the AI-based adaptive gamification category. These papers leveraged AI to make the gamification adaptive automatically based on the computer algorithms and data received from the target audience. Mittal et al. [80] designed an adaptive serious game in the area of blockchain to enhance the security knowledge-base of professionals and students. Their game can help improve the cybersecurity awareness of information management systems by allowing users to interact with the various components of the blockchain. This game offers to make use of players’ responses to adapt the gameplay by employing artificial intelligent game objects [80]. Moreover, Gasiba et al. [31] presented a platform where software developers can play a serious game (i.e., Capture-the-Flag) to raise their security awareness in secure coding. During the game, the players are assisted and provided hints through a virtual coach. A simple artificial intelligence engine based on the laddering technique for interviews is employed in the virtual coach to generate adaptive hints based on the given answers from the players. The authors also asserted that this virtual coach assisted “*the player in solving the challenge in a playful way and helps lower the frustration, increase the fun, and improve the learning effect during gameplay”*([83], p. 3).

Furthermore, Hatzivasilis et al. [79] proposed to leverage AI in their future studies to make gamification more human-centric. They presented an educational methodology for the dynamic adaptation of ISA programs such as gamification. Their methodology is based upon a data-driven model. They found that the dynamicity in gamification was mainly supported by an intelligent system to adapt the gamification based on the target group. Due to their data-driven design, they proposed to use machine learning to adapt the gamification in accordance with the participants' skills when a large number of profiles are collected from future iterations.

**Fig. 11:**
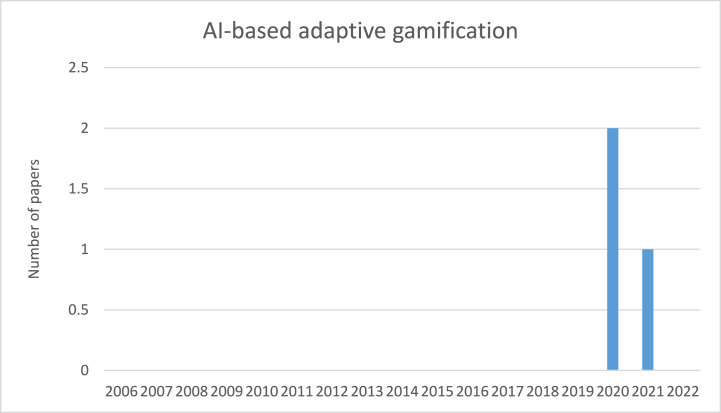
The number of papers using AI to make gamification user-tailored per year.

Fig. 11 depicts the number of papers that considered AI-based adaptive gamification per year. There is no published paper in this area before 2020. However, attention has been paid in terms of publications in this research area since 2020. By referring to the result from the previous section, 15 papers were classified as the adaptive gamification category, but only 3 of them are categorized in AI-based adaptive gamification. This means that 20 % of research concerning adaptive gamification benefited from AI. Furthermore, we did not find any paper that presents a method, framework, or model for designing and developing AI-based adaptive gamification within ISA programs.

## 5 Discussion

The main objective of this section is to discuss the findings of the systematic mapping study. Firstly, we discuss the results of each research question. Then we go over the threats to the validity of our study.

### 5.1 Discussion on the results of each research question

#### 5.1.1 Discussion on the result from RQ1

We limited the document types to journal papers and conference papers in the screening section. Fig. 2 shows that 54 % of the papers appeared in conference proceedings, and 46 % of the papers appeared in journals. Researchers can consider this when choosing the publication channel.

#### 5.1.2 Discussion on the results from RQ2

Gamification research in the domain of information security awareness has gained increasing attention starting in 2015. The decline in 2022 cannot violate this positive trend due to the time (i.e., early 2022) that this mapping study was conducted and many papers are yet to be published.

#### 5.1.3 Discussion on the results from RQ3

Evaluation research and validation research were the most frequent employed research types. This displays that solutions proposed by researchers in the field of gamification within ISA programs have been evaluated and implemented by researchers either in practice such as case studies or as an experiment in order to observe the impact of the proposed solution on employees' InfoSec awareness and behavior in addition to the benefits of and drawbacks of solutions. Having these outcomes and lessons learned provides a decent repository both for researchers and practitioners.

As presented in section 4.3, the most frequently reported contribution in the selected papers was the tool and there are a low number of papers that proposed frameworks and models. Having this in mind and considering the limited number of adaptive gamification and AI-based adaptive gamification employed by researchers, demonstrate the fact that it is essential to produce proper models and frameworks that could guide and help researchers in designing and developing gamification. This will be further discussed in the subsequent sections.

In section 4.3, we described that the major InfoSec topic used in these educational tools was phishing attacks which are in line with the topmost threats reported in recent years [3]. Although there are also a few papers about other subjects such as password policy and physical security in our list, it is important that researchers consider other InfoSec topics provided by NIST (2003) to have a comprehensive InfoSec learning system and support a wide range of employees with different roles and responsibilities.

#### 5.1.4 Discussion on the results from RQ4

According to the results from section 4.4, content gamification is utilized by researchers almost two times more than structural gamification, which means that content gamification (serious games), is more common in the domain of ISA programs.

One of the great advantages of structural gamification is flexibility, which enables organizations to conduct post-trainings without purchasing, or developing a new game for InfoSec training [20]. For post-training, organizations are able to reuse their current structural gamification just by changing the topic. For example, this adaptability provided by structural gamification can enable organizations to efficiently switch from one topic, such as phishing, to another, like password protection, simply by modifying the content within the existing structure. This approach not only reduces the cost and time associated with developing new training materials but also ensures a consistent training experience across different sessions.

However, the consistency and reusability that make structural gamification so effective can also lead to potential drawbacks, particularly in terms of user engagement. As users are exposed to the same graphics and interface repeatedly, the novelty may diminish, leading to decreased motivation and interest during post-trainings [20]. In contrast, content gamification offers a fresh experience for each training session, as it requires designing and developing unique gamified content tailored to specific topics. This variety can help maintain higher levels of engagement, as users encounter new challenges and interfaces with each session. Despite the engagement benefits of content gamification, the significant resources required for its continual development make it a more costly and labor-intensive option. Given the need for efficiency and cost management, we propose structural gamification over content gamification due to its strengths in reusability and economic significance.

However, a potential drawback of structural gamification is that it might become less engaging for users in post-trainings, possibly reducing their motivation. This could be due to the lack of changes in the game's graphics and interface, which might lead to a sense of repetitiveness [20]. In contrast, content gamification, which requires designing and developing gamification for each InfoSec topic separately, inherently avoids this issue by providing a fresh experience with each topic. While the potential for reduced user engagement in post-trainings is acknowledged, the economic benefits and ease of implementation offer effective reasons to prioritize structural gamification.

#### 5.1.5 Discussion on the results from RQ5

As presented in section 4.5, only 22 % of papers took adaptive gamification into consideration or employed adaptive gamification as an ISA tool, which is a limited share. Given that limit share, there is a gap in this area that should be filled out. Moreover, in section 4.5, we identified papers in which the authors expressed that merely conducting standard gamification without considering the target group is not possible to motivate and engage a wide range of participants, which is another indication of the necessity of employing adaptive gamification. Every organization consists of various types of employees with different roles and responsibilities. Therefore, the potential target group in gamification may range from simple users, who require basic information security knowledge on threats such as phishing, to security experts, who require hands-on experience in responding to information security incidents. This high diversity makes gamification quite a challenging task [79]. To deal with this challenge, it is imperative to adopt adaptive gamification in ISA programs that brings about benefits as follows.

• Increase efficiency and usability of gamification: The result of studies, which employed adaptive gamification in practice, revealed that user-tailored approaches could be of help to raise the effectiveness, coverage, engagement, and ability to reflect in the practice of gamification [84]. This approach has the ability to adapt to the time and resources required for the implementation of gamification within the business. Also, it plays a great role in mitigating information security risks of organizations by educating employees about information security threats [78].
• Elevate users' ISA: Through the use of adaptive gamification, we fulfill the three essential needs of users based on self-determination theory to maximize their intrinsic motivation [43]. Three basic psychological needs with self-determination theory are autonomy, which is a person's desire to self-organize their own activities; competence, which is a person's wish to have self-efficacy; and relatedness, which is a person's need for having the support and connection with others in the program [85]. For instance, by providing an opportunity for users that they can choose their desired InfoSec topic we fulfill autonomy, or by adjusting the game difficulty based on users' skills we fulfill competence. Studies have shown that by having these core needs satisfied individuals are more likely to engage in learning and display better performance [86,87] which in turn improves users' ISA [41].
• Enhance users' compliance with regard to InfoSec policies of the organization: Information security policy (ISP) is defined as *"established rules that provide guidance in the protection of an organization's assets"* [88]. These guidelines apply to all employees and external actors who work with organizations' information assets [88]. Employees' non-compliance with ISPs has been emphasized as a usual problem for many organizations [89]. To address this problem, researchers proposed ISA programs [29,90]. Bulgurcu et al. [90] stated that ISA can positively affect employees' attitudes and motivate them to follow company policies and procedures [90]. As we mentioned earlier, adaptive gamification can positively elevate users' ISA. Hence, employing gamification provides a means to both promote users' ISA and enhance their compliance with regard to ISPs of organizations [91].

In section 2.3, we mentioned that due to the dynamic nature of people's preferences and features, it is important to look at adaptive gamification as a continuous approach and not a one-time approach. However, according to the results in this study, only a few papers considered a continuous approach for adaptive gamification indicating a clear gap in the area. As a result, the ideal solution to maximize the aforementioned advantages of adaptive gamification is the attempt to continuously perform the adaption process (e.g., employing dynamic adaptive gamification).

#### 5.1.6 Discussion on the results from RQ6

In section 4.6, we only identified 3 papers in the AI-based adaptive gamification category out of 69 papers, and just one paper suggested the use of AI for future study. This identified limited number of papers demonstrates the clear gap in the area of AI-based adaptive gamification within ISA programs. This area not only needs more evaluation research and validation research to investigate the effectiveness of AI-based adaptive gamification and observe its impact on the ISA level of users, but also requires more solution of proposal research to cover the lack of method, framework, and model for designing and developing AI-based adaptive gamification within ISA programs.

As discussed in section 5.1.5, there was a limited study with adaptive gamification and dynamic adaptive gamification within ISA programs. Previous studies (e.g., Böckle et al.[42]; Khakpour & Colomo-Palacios [51]) suggested that the integration of AI or more specifically machine learning (ML) algorithms into gamification is a proper solution to make gamification adaptive and maintain its adaptiveness based on the target group. Therefore, artificial intelligence could be of help to fill the gaps in adaptive gamification and dynamic adaptive gamification within ISA programs.

Machine learning and artificial intelligence provide great support in making gamification adaptive to a person's behavior in a dynamic manner [79]. As we mentioned before, this task requires frequent assessments with users to receive their feedback and update the gamification accordingly, which might be cumbersome in practice. AI can be helpful to build a data-driven model to perform this process [79]. These technologies help in gathering data from users at a fast pace which saves both time and money due to omitting manual efforts. It provides genuine and reliable outcomes without human biases. Moreover, these techniques can also serve as a powerful tool to simply analyze and interpret data. As a result, integrating AI into gamification could assist researchers to probe and understand how they can design gamification in a more user-centered way to increase its efficiency [84], which in turn enhances users' ISA [41] and compliance with ISPs [90].

It is worth mentioning that developing machine learning models for gamification requires a high level of knowledge and skills to ensure their efficiency and reliability [92], this could be one of the reasons for the existence of the gap in AI-based adaptive gamification gap within ISA programs. We believe that one way to bridge this gap is to utilize already built machine learning models proposed by researchers (e.g., Barata et al. [93]; Lopez & Tucker [52]) to adapt gamification based on the target group in ISA programs.

### 5.2 Threats to validity

The threats to the validity of this mapping study are discussed as follows.

• Study Selection Validity: This category includes threats that can be identified during the initial two phases of the systematic mapping study (i.e., conducting the search and paper screenings), such as the selection of digital libraries and search string construction [94]. We had some biases in this phase including the selection of databases (only Scopus and the Web of Science), inclusion criteria (i.e., limiting to journal papers and conference papers), and the search strings (absence of some synonyms). For instance, some additional search strings could have been used for the literature search. Therefore, there is a possibility that we missed or overlooked some studies. However, we believe that the selected databases and chosen research strings cover the most relevant published literature in the domain of gamification and ISA programs. Furthermore, the perspective from NIST [27] has been used as a foundation for the different topics in ISA in this study. We acknowledge that other sources could have served as alternative bases for the different topics in ISA. In addition, we could have included more research questions to address other topics (such as, scoring/grading aspect in gamification) in this mapping study. However, we limit the scope of this mapping study to the proposed six questions. Addtionally, the included articles in this literature search are up until March 2022. This means that articles published after March 2022 have not been included in this study.
• Data Validity: Threats in this category can be specified during the last two phases of the systematic mapping study, which are the classification scheme, data extraction and analysis [94]. Some examples are misclassification of studies, lack of statistical analysis, bias in classification schema, researcher bias, etc. Some bias may exist in making the selection of our classification schemes. To cope with this, we used some existing classifications for the research type facet and the research contribution facet proposed by other researchers such as Wieringa et al. [55] and then tried to adapt them to fit our study. Other classification facets such as gamification type are based on experts' opinions in the field of gamification such as Dubey [38] and by conducting the literature review. Another bias is the way we perform data extraction and analysis. Sometimes it was difficult to decide which category was proper for one specific paper. For instance, in the research type facet, we were not sure which category (e.g., evaluation research or validation research) was the best choice for some papers. Thus, there is a probability that other researchers would perform this differently. To address this issue, we benefited from some quality criteria such as questions proposed by Wieringa et al. [55] in the research type facet to avoid the data extraction bias. A common view regarding mapping studies is that normally just by reading abstracts it is possible to conduct the study. This fact is likely if the abstract of papers is structured in a good way. However, we sometimes found the data extraction difficult, because some abstracts were misleading and lacked important information. Therefore, we read other parts of the papers as well such as conclusion and introduction to ensure the accuracy of our data extraction.
• Research validity: This category includes threats that can be identified in all phases of the study, and concerning the overall research design [94]. Some of these threats include coverage of research questions, generalizability, etc. To cope with the threat of coverage of research questions, we attempted to motivate each research question well and opt for a proper classification scheme to find the best possible answer to each research question. Generalizability in a mapping study is referred to whether the results of the study are generalizable or not [94]. The results drawn from this study only concern this systematic mapping study so this threat does not apply to our study.

## 6 Conclusion

This study aimed to offer a comprehensive overview of the existing literature on gamification and ISA programs. The main objective was to identify trends, gaps, and patterns within this research field to guide future research. To achieve this, a systematic mapping study followed a method proposed by Petersen et al. [26] was carried out. Out of 1499 papers from the initial search, 69 papers were selected and classified according to the following criteria: document type, year of publication, research type, research contribution, gamification type, gamification in terms of adaptivity based on the target group, and gamification in terms of the use of AI to make it user-tailored.

The results indicated that published papers in this area are split between journals and conference papers with a higher proportion published in conference proceedings. Regarding the publication trend, from 2015 to 2022, gamification within ISA programs has come across to researchers' attention. The identified two main research types were evaluation research and validation research and the vast majority of the contribution type was tools. These tools mostly were gamification in ISA programs to address users' lack of InfoSec knowledge. Among proposed gamification, many of them were content gamification (serious games), however, by reviewing the selected papers, we think that structural gamification could be a better choice due to its reusability function saving both money and time.

This study has shown that there is a clear gap in the use of adaptive gamification and dynamic adaptive gamification. This lack of the use of adaptive gamification would have some negative influences on the efficiency of applied gamification within ISA programs, which would possibly lead to a decline in employees’ ISA and their ISP compliance. Artificial intelligence has been suggested in previous studies (e.g., Böckle et al. [42]; Khakpour & Colomo-Palacios [51]) as a proper solution to make gamification adaptive based on the target group and maintain its adaptiveness during the time. However, only three papers are classified as the AI-based adaptive gamification category in this study, which indicates a clear gap in integrating AI into gamification within ISA programs.

These identified gaps also open some opportunities for future research in gamification within ISA programs. Firstly, there is a need for more frameworks and models to guide research regarding gamification within ISA programs. For instance, how dynamic adaptive gamification can be designed to enhance users’ ISA more efficiently. Secondly, it would be interesting to explore how to design structural gamification within ISA programs to have the features of reusability, the ability to use various information security topics without redesigning the game. This type of structural gamification would have a great role in saving time and resources for organizations in conducting ISA programs. Thirdly, researchers can also expand the scope of this mapping study to study other interesting aspects regarding gamification within ISA programs, such as, user experience design, ethical considerations, and so on. Last but not least, researchers can further look into how AI can be employed to make gamification adaptive within ISA programs. This would require developing machine learning models by considering theories and frameworks such as self determination theory or employing existing reliable machine learning models to personalize gamification.

## Data availability statement

Data included in article/supplementary material is referenced in the article.

## CRediT authorship contribution statement

**Omid Pahlavanpour:** Writing – review & editing, Writing – original draft, Methodology, Investigation, Formal analysis, Data curation, Conceptualization. **Shang Gao:** Writing – review & editing, Writing – original draft, Validation, Supervision, Methodology, Investigation, Conceptualization.

## Declaration of competing interest

The authors declare the following financial interests/personal relationships which may be considered as potential competing interests:The corresponding author of this manuscript is an associate editor for Heliyon (Information science section, and computer science section).

## Figures and Tables

**Table:** 

References	Title	Document Type	Research Type	Contribution type	RQ4	RQ5	RQ6
[77]	PROTECT – An Easy Configurable Serious Game to Train Employees Against Social Engineering Attacks	Conference Paper	Evaluation research	Tool	0	1	0
(T. E. [31])	Cybersecurity challenges in industry: Measuring the challenge solve time to inform future challenges	Journal Paper	Evaluation research	Method-Tool- Guideline/Open item	0	1	0
(T. [95])	CyberSecurity Challenges for Software Developer Awareness Training in Industrial Environments	Conference Paper	Evaluation research	Tool- Guideline/Open item	0	1	0
[83]	Sifu - a cybersecurity awareness platform with challenge assessment and intelligent coach	Journal Paper	Evaluation research	Tool-Method	0	1	1
[97]	Learning security through computer games: Studying user behavior in a real-world situation	Conference Paper	Evaluation research	Tool-Model	0	0	0
[98]	Teaching johnny not to fall for phish	Journal Paper	Evaluation research	Tool	0	0	0
[99]	A Serious Game for Eliciting Social Engineering Security Requirements	Conference Paper	Evaluation research	Tool	0	0	0
[100]	PERSUADED: Fighting Social Engineering Attacks with a Serious Game	Conference Paper	Evaluation research	Tool	0	0	0
[101]	Effectiveness of and user preferences for security awareness training methodologies	Journal Paper	Evaluation research	Guideline/Open item	0	0	0
[102]	Battlespace next™: Developing a serious game to explore multi-domain operations	Journal Paper	Evaluation research	Framework-Tool	0	0	0
[103]	Design and evaluation of an augmented reality game for cybersecurity awareness (CybAR)	Journal Paper	Evaluation research	Tool	0	0	0
[104]	Identification of Unintentional Perpetrator Attack Vectors using Simulation Games: A Case Study	Conference Paper	Evaluation research	Tool	0	0	0
[105]	Sherlocked: A Detective-Themed Serious Game for Cyber Security Education	Conference Paper	Evaluation research	Tool	0	0	0
[78]	Persona-centered information security awareness	Journal Paper	Evaluation research	Method	N	1	0
[74]	Growing a cyber-safety culture amongst school learners in South Africa through gaming	Journal Paper	Evaluation research	Method- Guideline/Open item	N	N	0
[20]	A serious game for healthcare industry: Information security awareness training program for Hospital Universiti Kebangsaan Malaysia	Journal Paper	Evaluation research	Tool	1	1	0
[71]	Control-Alt-Hack: The design and evaluation of a card game for computer security awareness and education	Conference Paper	Evaluation research	Tool	1	0	0
[106]	Shell we play A game? CTF-as-a-service for security education	Conference Paper	Evaluation research	Tool	1	0	0
[107]	Phish Derby: Shoring the Human Shield Through Gamified Phishing Attacks	Journal Paper	Evaluation research	Tool	1	0	0
[31]	Cybersecurity Awareness Platform with Virtual Coach and Automated Challenge Assessment	Conference Paper	Evaluation research	Tool-Method	0	1	1
[108]	Teaching phishing-security: Which way is best?	Conference Paper	Evaluation research	Guideline/Open item	N	N	0
[109]	Using game design to teach informatics and society topics in secondary schools	Journal Paper	Evaluation research	Guideline/Open item	N	N	0
[85]	Implement a Model for Describing and Maximising Security Knowledge Sharing	Conference Paper	Evaluation research	Tool	1	1	0
[82]	Shuffle, cut, and learn: Crypto go, a card game for teaching cryptography	Journal Paper	Evaluation research	Tool	1	0	0
[110]	Choose your own training adventure: designing a gamified SETA artefact for improving information security and privacy through interactive storytelling	Journal Paper	Evaluation research	Tool- Guideline/Open item	1	0	0
[72]	Assessing the effects of gamification on enhancing information security awareness knowledge	Journal Paper	Evaluation research	Guideline/Open item	1	0	0
[70]	Raising secure coding awareness for software developers in the industry	Conference Paper	Experience Papers	Method	N	N	0
[69]	It's all fun and games, and some legalese: Data protection implications for increasing cyber-skills of employees through games	Conference Paper	Opinion paper	Guideline/Open item	N	N	0
[68]	The Human Factor in the Social Media Security – Combining Education and Technology to Reduce Social Engineering Risks and Damages	Journal Paper	Opinion paper	Guideline/Open item	N	N	0
[111]	Defending against phishing attacks: taxonomy of methods, current issues and future directions	Journal Paper	Philosophical Papers	State of Knowledge	N	N	0
[112]	Coronavirus social engineering attacks: Issues and recommendations	Journal Paper	Philosophical Papers	State of Knowledge- Guideline/Open item	N	N	0
[66]	Taxonomy of Cybersecurity Awareness Delivery Methods: A Countermeasure for Phishing Threats	Journal Paper	Philosophical Papers	State of Knowledge	N	N	0
[67]	Cybersecurity awareness: A critical analysis of education and law enforcement methods	Journal Paper	Philosophical Papers	State of Knowledge- Guideline/Open item	N	N	0
[80]	Cybersecurity Enhancement through Blockchain Training (CEBT) – A serious game approach	Journal Paper	Solution proposal	Tool	0	1	1
[113]	Changing users' security behaviour towards security questions: A game based learning approach	Conference Paper	Solution proposal	Tool	0	0	0
[64]	Self-efficacy-based game design to encourage security behavior online	Conference Paper	Solution proposal	Tool	0	0	0
[84]	Designing Serious Games for Cyber Ranges: A Socio-Technical Approach	Conference Paper	Solution proposal	Framework	N	1	0
[65]	Cyber security games: A new line of risk	Conference Paper	Solution proposal	Framework	N	N	0
[114]	A case for societal digital security culture	Conference Paper	Solution proposal	Guideline/Open item	N	N	0
[115]	Building confidence not to be phished through a gamified approach: Conceptualising user's self-efficacy in phishing threat avoidance behavior	Conference Paper	Solution proposal	Model	N	N	0
[116]	Designing Analytic Serious Games: An Expert Affordance View on Privacy Decision-Making	Conference Paper	Solution proposal	Guideline/Open item	N	N	0
[117]	The use of computer games for teaching and learning cybersecurity in higher education institutions	Journal Paper	Solution proposal	Framework- State of Knowledge	N	N	0
[79]	Modern aspects of cyber-security training and continuous adaptation of programmes to trainees	Journal Paper	Solution proposal	Method	N	1	0
[118]	Cyber security training and awareness through game play	Conference Paper	Validation research	Tool	0	1	0
[119]	Riskio: A Serious Game for Cyber Security Awareness and Education	Journal Paper	Validation research	Tool	0	1	0
[120]	Anti-Phishing Phil: The design and evaluation of a game that teaches people not to fall for phish	Conference Paper	Validation research	Tool	0	0	0
[121]	Raising information security awareness in digital ecosystem with games - A pilot study in Thailand	Conference Paper	Validation research	Guideline/Open item	0	0	0
[76]	Phishing threat avoidance behaviour: An empirical investigation	Journal Paper	Validation research	Tool	0	0	0
[81]	"smells Phishy?": An educational game about online phishing scams	Conference Paper	Validation research	Tool	0	0	0
[122]	Game based cybersecurity training for High School Students	Conference Paper	Validation research	Method-Tool	0	0	0
[91]	Autonomy Motivators, Serious Games, and Intention Toward ISP Compliance	Journal Paper	Validation research	Guideline/Open item	0	0	0
[123]	A data-driven security game to facilitate information security education	Conference Paper	Validation research	Tool	0	0	0
[124]	Decision-making and biases in cybersecurity capability development: Evidence from a simulation game experiment	Journal Paper	Validation research	Tool	0	0	0
[125]	Investing in Prevention or Paying for Recovery - Attitudes to Cyber Risk	Conference Paper	Validation research	Tool	0	0	0
[75]	PenQuest: a gamified attacker/defender meta model for cyber security assessment and education	Journal Paper	Validation research	Model-Framework-Tool	0	0	0
[126]	Raising Awareness about Cloud Security in Industry through a Board Game	Journal Paper	Validation research	Tool	0	0	0
[127]	Operation digital Ant: A serious game approach to collect insider threat scenarios and raise awareness	Conference Paper	Validation research	Tool	0	0	0
[128]	HATCH: Hack and trick capricious humans – A serious game on social engineering	Conference Paper	Validation research	Tool	0	0	0
[129]	On the design of playful training material for information security awareness	Conference Paper	Validation research	Method-Tool	0	0	0
(T. [130])	Design of secure coding challenges for cybersecurity education in the industry	Conference Paper	Validation research	Guideline/Open item	N	N	0
[131]	Raising Security Awareness Using Cybersecurity Challenges in Embedded Programming Courses	Conference Paper	Validation research	Method- Guideline/Open item	N	N	0
[33]	Gamification of information security awareness and training	Conference Paper	Validation research	Tool	1	1	0
[63]	Playing safe: A prototype game for raising awareness of social engineering	Conference Paper	Validation research	Tool	1	0	0
[62]	Gamification Techniques for Raising Cyber Security Awareness	Conference Paper	Validation research	Tool	1	0	0
[132]	The Good, the Bad and the Ugly: A Study of Security Decisions in a Cyber-Physical Systems Game	Journal Paper	Validation research	Tool	1	0	0
[133]	Malware Awareness Tool for Internet Safety using Gamification Techniques	Conference Paper	Validation research	Tool	1	0	0
[73]	CySecEscape 2.0-A virtual escape room to raise cybersecurity awareness	Journal Paper	Validation research	Tool	1	0	0
[134]	A game design framework for avoiding phishing attacks	Journal Paper	Validation research	Model	N	N	0
[135]	A video game for cyber security training and awareness	Journal Paper	Validation research	Tool	1	1	0

## Appendix A The Filled Data Extraction Form

RQ4: Structural gamification = 1, Content gamification = 0, None: N.
RQ5: Adaptive gamification = 1, Non-adaptive gamification = 0, None = N.
RQ6: AI-based adaptive gamification = 0, Non-AI-based adaptive gamification = 1.
